# Poly (Orange CD) sensor for paracetamol in presence of folic acid and dopamine

**DOI:** 10.1038/s41598-021-01311-5

**Published:** 2021-11-16

**Authors:** S. D. Sukanya, B. E. Kumara Swamy, J. K. Shashikumara, S. C. Sharma, S. A. Hariprasad

**Affiliations:** 1 Department of P.G. Studies and Research in Industrial Chemistry, Kuvempu University, Jnana Sahyadri, Shankaraghatta, Shivamogga, Karnataka 577451 India; 2 National Assessment and Accreditation Council (Work Carried Out as Honorary Professor), Jain University, Bangalore, Karnataka 560 069 India; 3 School of Energy Science and Engineering, Indian Institute of Technology Guwahati, Guwahati, India; 4 Jain University, Bangalore, Karnataka 560 069 India

**Keywords:** Biochemistry, Chemistry, Physics

## Abstract

In the present work, Orange CD was chosen as an intriguing modifier for the electropolymerization on the surface of CPE by the CV technique. A novel, sensitive, and cost-effective poly (Orange CD) MCPE (PoOCD/MCPE) sensor was utilized for the selective detection of paracetamol (PA) in 0.2 M phosphate buffer solution (PBS) of pH 7.4. The oxidation peak current of PA was vastly enhanced at the sensor. The scan rate study is suggested that electro-oxidation of PA was adsorption-controlled. The pH study testifies the redox pathways transport with the same quantity of electrons and protons. The detection limit of PA is found to be 2.64 µM. DPV results show that substantial peak separation between PA, folic acid (FA), and dopamine (DA) could be facilitating their individual and simultaneous determination on the sensor. The decorated sensor demonstrates high sensitivity, stability, reproducibility, repeatability and has been successfully exploited for the detection of PA in a tablet with promising results.

## Introduction

Paracetamol (PA) is one of the most extensively used analgesics and antipyretic drugs in clinical practice^1,2^. It is a very effective agent recommended for mild to moderate pain alleviation such as flu-induced fever, migraine, arthritis, and extenuates pain (headache, toothache, joint, muscular, chronic, postoperative)^3,4^. PA relieves pain by inhibiting prostaglandin synthesis in the central nervous system, and it also relieves fever by sedating the hypothalamus heat-regulating center^5^. PA is easily degraded by glucuronidation and sulfation into inactive metabolites, which are excreted in the urine, with just 5% of PA remaining unaltered^6^. In general, PA is known to have an excellent safety profile at approved therapeutic doses. But, its toxic metabolite accumulation in case of overdosing and chronic use lead to harmful side effects such as liver problem, kidney damages, trembling, nervousness, seizures, insomnia and nausea and even death^7–10^. Therefore, developing a simple, fast response, economical, sensitive, accurate, and reliable detection method for the assessment of PA is highly demanded in the medical field. There are lots of methods like capillary electrophoresis^11^, titrimetry^12^, SEC, LC–MS, HPLC^13–15^, chemiluminescence^16^, spectrofluorimetric^17^, and spectrophotometry^18–20^ which have been availed for the assessment of PA. Among all these methods, the electrochemical method stands out with its simplicity, sensitivity, selectivity, modest and fast response.

Folic acid (FA) is a water-soluble vitamin B_9_ and also known as folacin that helps the growth of healthy new cells especially during pregnancy and controls the generation of ferrohaeme. FA is involved in a variety of biological tasks related to cell metabolism, including DNA replication, repair, and methylation, as well as the production of nucleotides, vitamins, and amino acids. Deficiency of FA causes anemia, leucopoenia, devolution of mentality, neurosis and also increases the chances of heart attack and stroke^21–24^. Dopamine (DA) is the neurotransmitter involved in the functioning of the central nervous system. DA is also utilized as an injectable medicine that stimulates the sympathetic nervous system, causing effects such as increased blood pressure and heart rate. Deficiency of DA may cause disorders like Parkinson’s disease, Schizophrenia, Alzheimer’s disease, and HIV infection^25–28^. When used for a long time, nonsteroidal anti-inflammatory agent like PA can prevent FA from being absorbed by the human being. The simultaneous measurement of PA and FA is particularly relevant since PA enhances the need for FA. The usage of PA protects dopaminergic neurons against oxidative stress damage produced by acute exposure to increased amounts of DA, according to in vitro studies. Furthermore, prolonged PA use in in vivo model has been shown to dramatically lower DA levels. Selective or simultaneous detection of PA, FA, and DA have been achieved by voltammetric method due to their electroactive natures^29–32^. In the electrochemical sensor field, electropolymerized MCPE has made a great contribution to the determination of biomolecules because of their good stability, homogeneity, strong adhesion of polymer film onto the electrode surface, more active sites, fine reproducibility, fine resolution voltammogram, low-cost, and easy preparation method^33–36^. As redox dyes are artificial electron donatives, they are effective to undergo electropolymerization and produce stable redox-active films^37,38^.

**Scheme 1.**
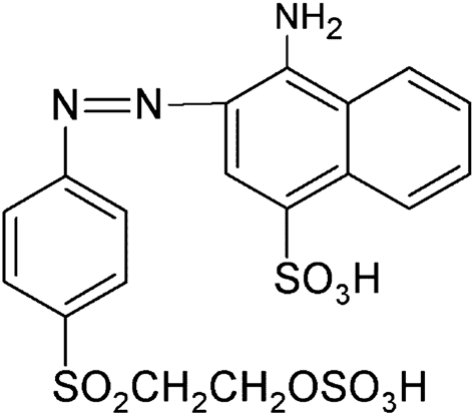
Structure of Orange CD.

Present work explores, less studied Orange CD (Scheme 1) dye^39^ as a modifier for the electropolymerization on the CPE surface by CV technique. The performance of PoOCD/MCPE was assessed for the sensitive, selective determination of PA and simultaneous determination of PA, FA, and DA in biological pH 7.4. The sensor displayed higher electrocatalytic activity, as well as a low detection limit and large linear ranges for PA resolution. The practical applicability of the sensor has been tested by determining PA in tablets successfully. This work is intended to pave the way for the development of more efficient, dependable, and generally affordable sensors.

## Experimental

### Materials and instrumentation

All analytical grade chemicals such as PA (M_wt_ = 151.16 gmol^−1^, purity 99%), FA (M_wt_ = 441.40 gmol^−1^, purity 99.5%), DA (M_wt_ = 189.64 gmol^−1^, purity 99%), graphite powder, Na_2_HPO_4_, and NaH_2_PO_4_·H_2_O was procured from Merck Chemicals (Mumbai, India) and Orange CD dye from Astik Dyestuff Pvt. Ltd (Gujarat, India). Stock solutions of orange CD, PA, FA, and DA with a concentration of 25 × 10^–4^ M were prepared in double-distilled water (DDW). The 0.2 M PBS was prepared by Na_2_HPO_4_ and NaH_2_PO_4_·H_2_O.

Voltammetric measurements were conducted in CHI-660c model (CH Instrument-660 electrochemical workstation, USA) analytical system. An electrochemical cell (25 ml) consisting of saturated Calomel electrode (Equip-tronics, Mumbai), platinum wire (Equip-tronics, Mumbai)) and bare CPE or PoOCD/MCPE, were acted as a reference, counter, and working electrodes respectively at room temperature.

### Preparation of paracetamol tablet sample

In a mortar, a 500 mg of Calpol pill was acquired from local drug stores (Shivamogga, India) was finely pulverized. In a 100 ml flask, an adequate amount of homogenous white powder was dissolved in water. The solution was thoroughly agitated to get the appropriate concentration before being utilized in pharmaceutical sample analysis.

### Working electrode construction

The bare CPE was prepared as described in the literature^40^. The PoOCD/MCPE was constructed by dipping bare CPE into 1 mM aqueous Orange CD with NaOH (0.1 M) as a supporting electrolyte. The electrochemical polymerization was performed at the potential between − 0.6 and 1.6 V with a scan rate (SR) of 100 mVs^−1^ using ten cycles. Then obtained electropolymerized electrode was rinsed in the DDW to eliminate unreacted molecules.

## Results and discussion

### Electrochemical polymerization of Orange CD on bare CPE

**Figure 1:**
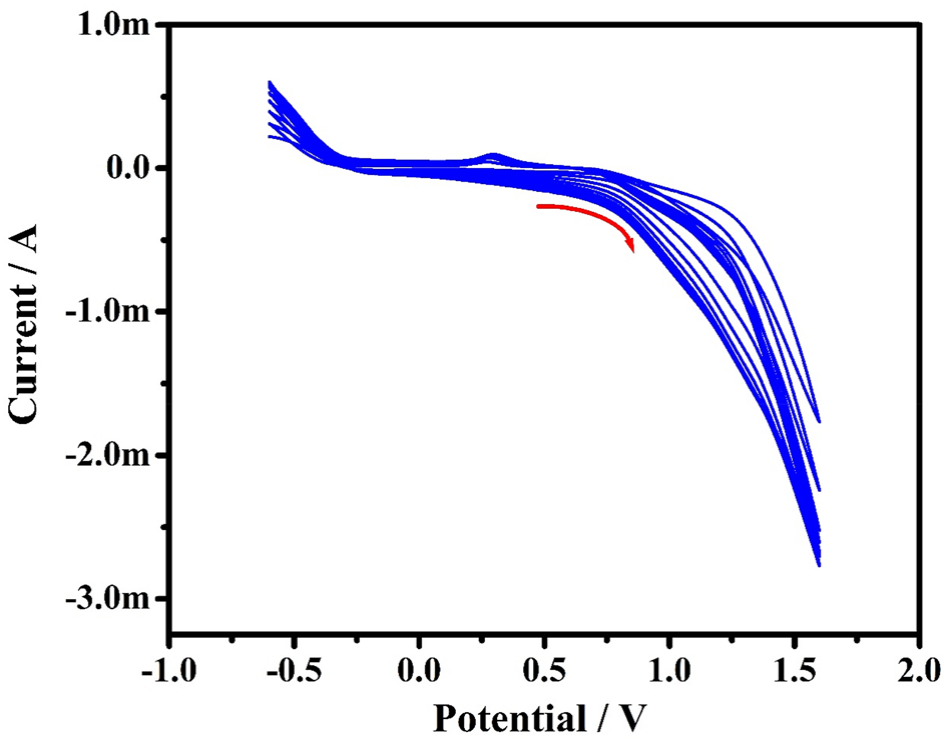
CVs of construction of PoOCD/MCPE with 0.1 M NaOH for ten cycles at SR of 100 mVs^−1^.

**Figure 2:**
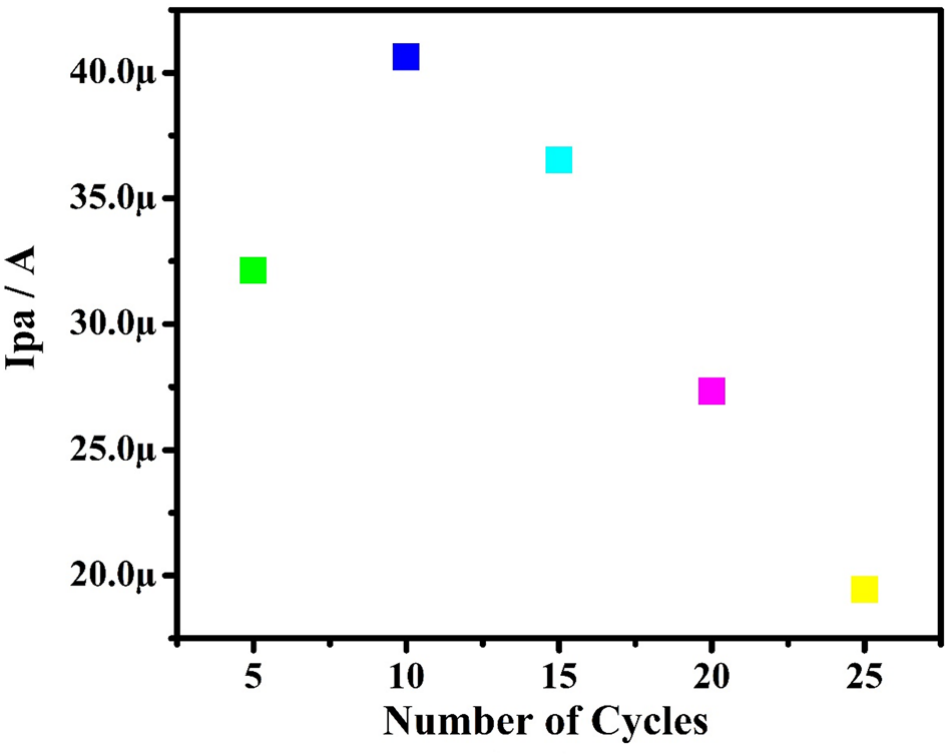
Graph of Ipa vs number of voltammetric scans.

Figure 1 shows the CVs of electrochemical polymerization of 1 mM aqueous orange CD with NaOH (0.1 M) on the bare CPE surface in the potential cycling between − 0.6 and 1.6 V with SR of 100 mVs^−1^ using ten cycles. The examination of voltammograms by gradually increasing the progressing electropolymerization procedure reveals accumulation and growth of orange CD film on the surface of bare CPE^41^. The polymer film thickness affects the electrochemical response of the modified electrode. The film thickness was easily managed by regulating the number of voltammetric scans from 5 to 25 during electropolymerization. The experimental results analogous to it were obtained for the PA as shown in Fig. 2. As the current response achieves a maximum at ten multiple cycles, the optimum cycle number of ten was selected for the construction of PoOCD/MCPE and further voltammetric measurements.

### Characterization of PoOCD/MCPE

**Figure 3:**
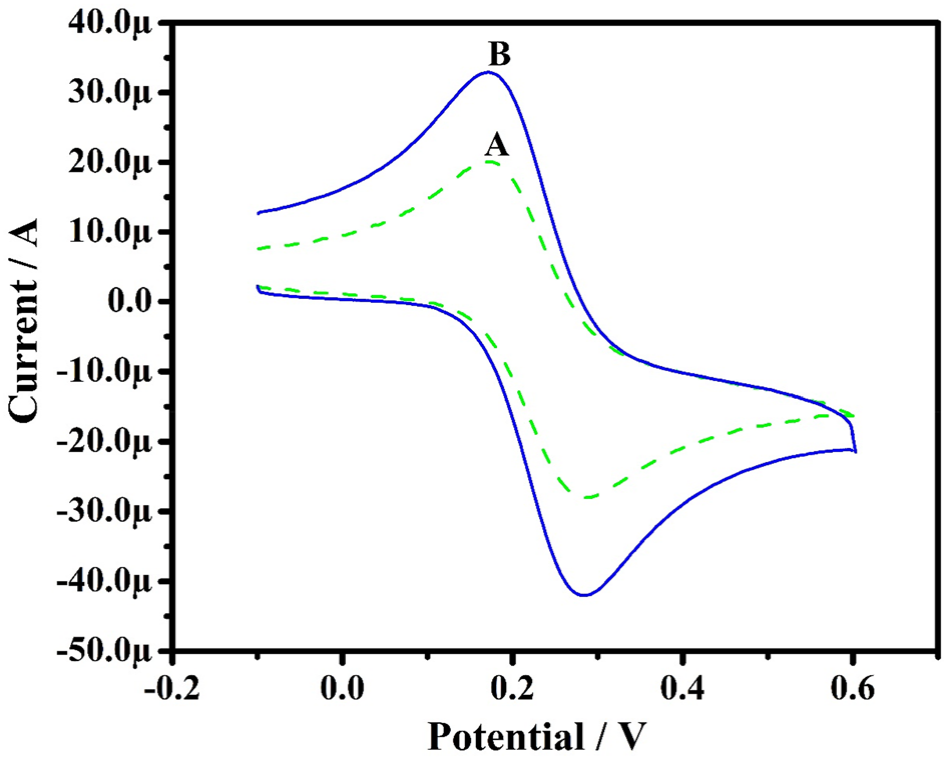
CV results of K_4_ [Fe (CN)_6_] at bare CPE (A) and PoOCD/MCPE (B) at a SR 100 mVs^−1^.

For investigation of electrocatalytic activity of the MCPE, a potassium ferrocyanide system was used. Figure 3 displays the electrochemical activity of K_4_[Fe(CN)_6_] (freshly prepared) at bare CPE (A) and PoOCD/MCPE (B) containing 1 M KCI as supporting electrolyte obtained at an SR of 100 mVs^−1^ was recorded by CV method. The small redox peak current signal corresponds to bare CPE while PoOCD/MCPE shows enhanced peak current showing the dramatic increase in the rate of electron transfer^33^. According to Randles–Sevick’s Eq. (1), the electrocatalytic surface area of both bare CPE and MCPE was calculated^42^.

1 $$ {\text{Ip}} = \left( {2.69 \times 10^{5} } \right){\text{n}}^{3/2} {\text{AD}}^{1/2} \nu ^{1/2} {\text{C}} $$

The area of bare CPE (0.0295 cm^2^) is less than PoOCD/MCPE area (0.0499 cm^2^) which indicates that Orange CD acts as an effective modifier contributing a large surface and promotes the electron transfer between the electrode and the solution.

**Figure 4:**
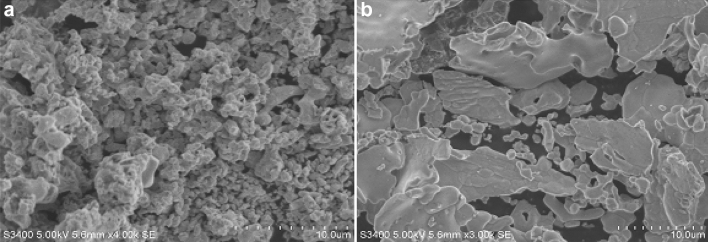
SEM of bare CPE (**a**) and PoOCD/MCPE (**b**).

The surface morphological features of bare CPE and PoOCD/MCPE were characterized by SEM. The SEM of bare CPE (Fig. 4a) appears to be a rough surface with irregularly shaped and PoOCD/MCPE (Fig. 4b) appears to be a smooth with consistent ordering of the polymer film of Orange CD on the CPE surface. The remarkable distinction in the surface structure of both electrodes confirms the remarkable modification of the CPE surface by electropolymerized Orange CD.

### Voltammetric measurements

**Figure 5:**
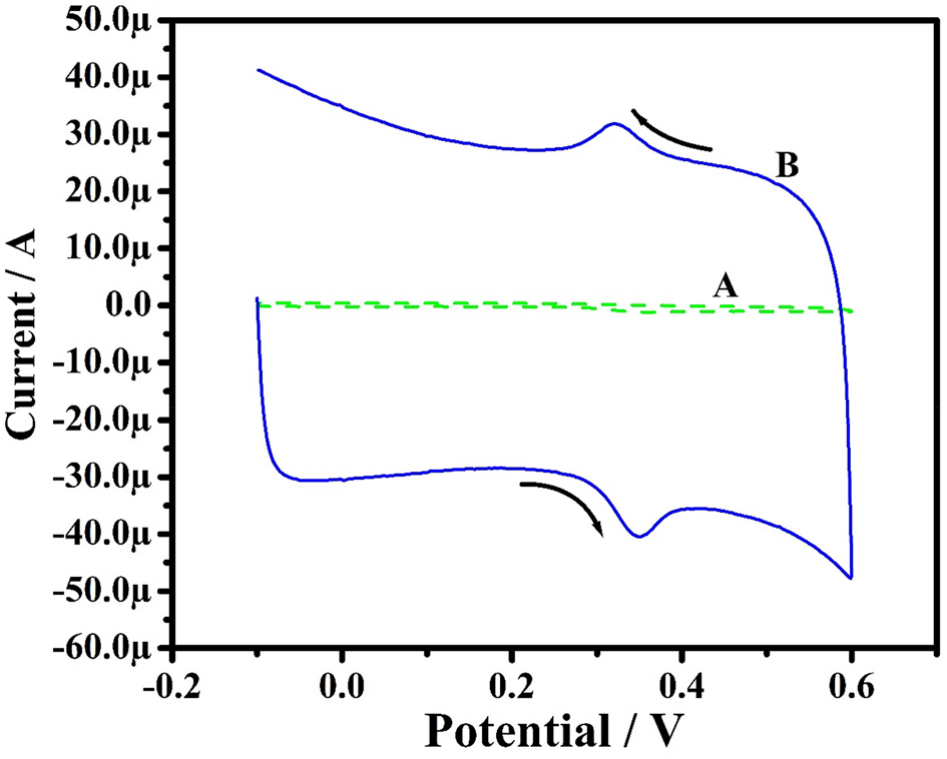
CVs for PA (0.1 mM) in 0.2 M PBS (pH 7.4) at bare CPE (A) and PoOCD/MCPE (B) at SR 100 mVs^−1^.

The electrochemical response of PA was studied on the bare CPE (A) and PoOCD/MCPE (B) in 0.2 M PBS (pH 7.4) at an SR 100 mVs^−1^ by CV method as displayed in Fig. 5. An irreversible voltammogram was obtained at bare CPE for PA with an anodic peak potential of 0.357 V indicating the poor response as well as the occurrence of only oxidation. But at the same condition, PoOCD/MCPE exhibited a significant increase in the current signals giving a sharp reversible voltammogram. The anodic and cathodic peak potential for PA were found to be 0.349 V and 0.320 V respectively reveals the occurrence of both oxidation and reduction at proposed PoOCD/MCPE.

**Figure 6:**
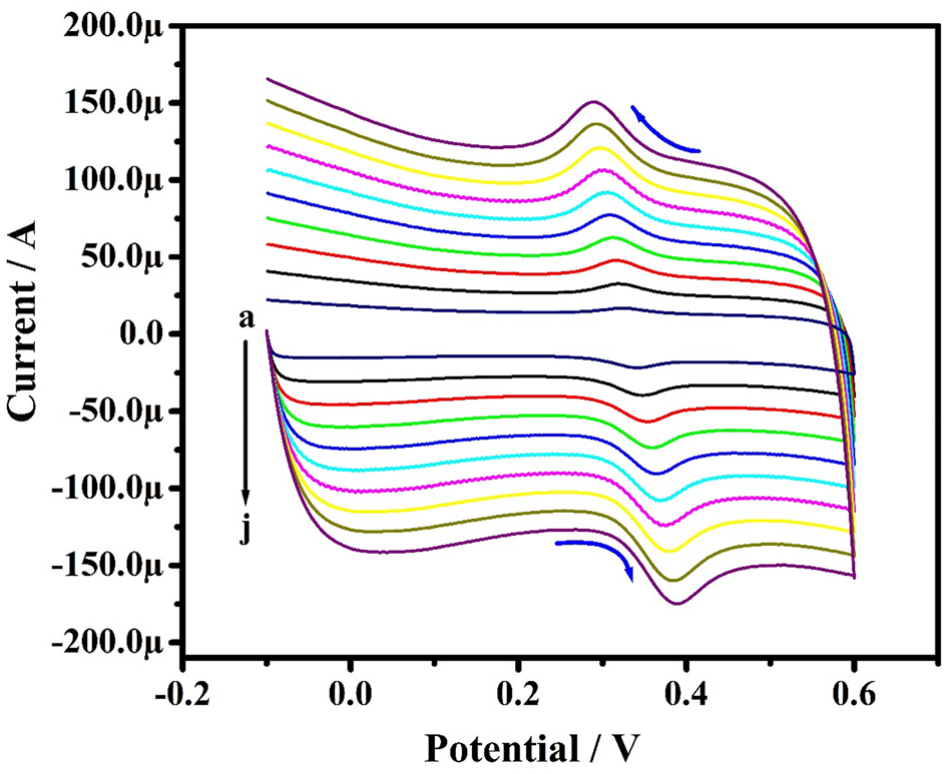
CVs obtained for PA (0.1 mM) at PoOCD/MCPE with various SR (50–500) mVs^−1^ in PBS (0.2 M, pH 7.4).

**Figure 7:**
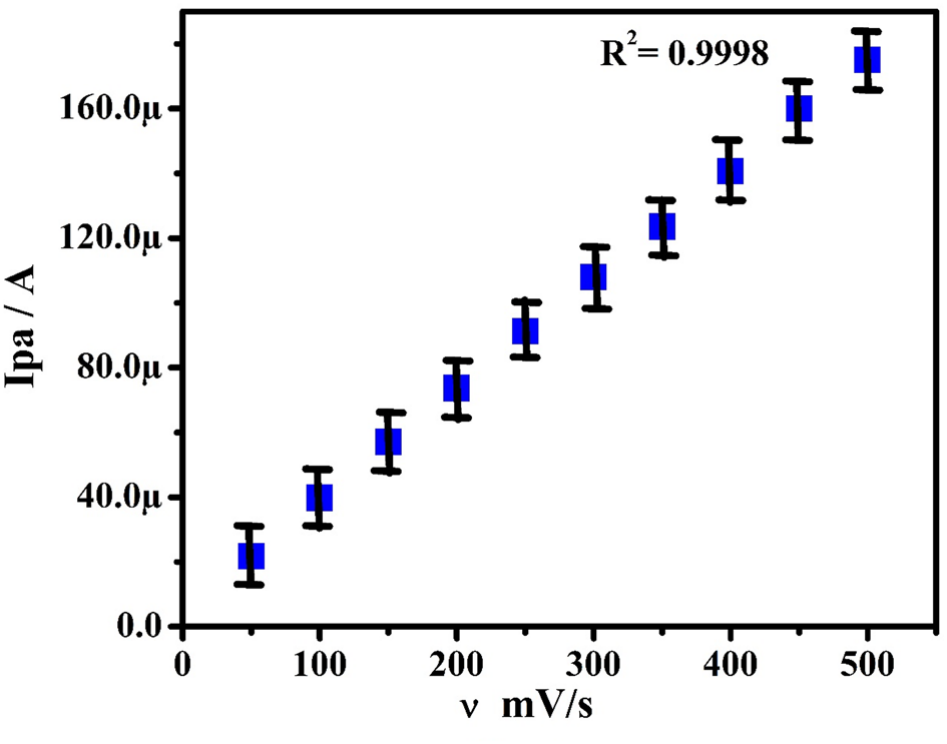
Graph of Ipa vs SR of PA (0.1 mM) in PBS (0.2 M, pH 7.4).

**Figure 8:**
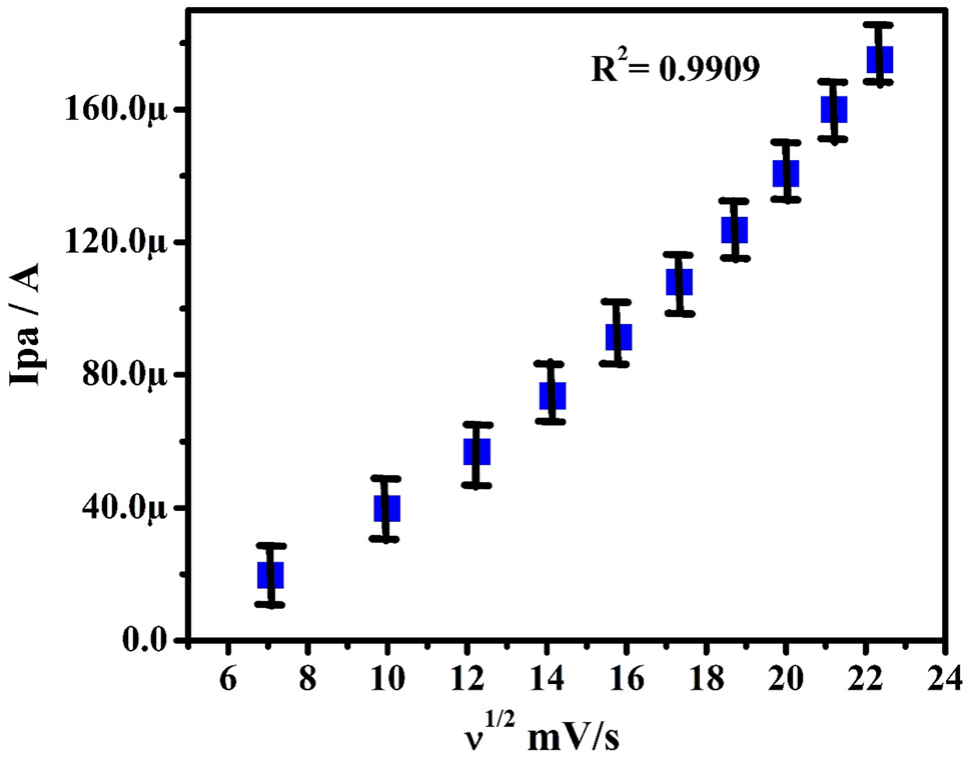
Graph of Ipa vs square root of SR of PA (0.1 mM) in PBS (0.2 M, pH 7.4).

**Figure 9:**
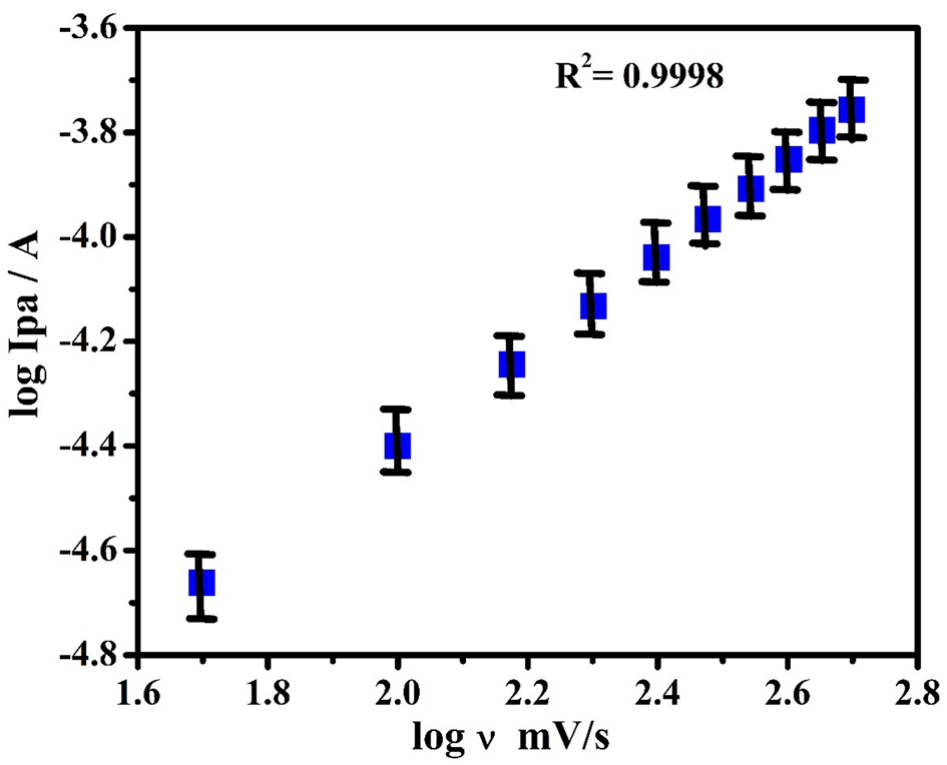
Graph of log Ipa vs log SR of PA (0.1 mM) in PBS (0.2 M, pH 7.4).

The impact of potential scan rate (SR) for the electrochemical studies of 0.1 mM PA in 0.2 M PBS (pH 7.4) from 50 to 500 mVs^−1^ was investigated by the CV method at PoOCD/MCPE as depicted in Fig. 6. It is found that the redox peak currents rise with rising scan rates. The electrode phenomenon is controlled by adsorption at PoOCD/MCPE for PA as deduced from the good linearity with regression equations Ipa (µA) = 0.34 ʋ (mV/s) + 5.56 (µA) (R^2^ = 0.9998), Ipa (µA) = 1.02 ʋ (mV/s) − 6.39 (µA) (R^2^ = 0.9909) and Ipa (µA) = 0.90 log ʋ (V/s) − 6.21 (µA) (R^2^ = 0.9998) of the Ipa vs SR (Fig. 7), Ipa vs square root of SR (Fig. 8) and log Ipa vs log SR (Fig. 9) plots respectively^43,44^. The heterogeneous rate constant (k^0^) were estimated for such voltammograms whose ∆Ep (experimental peak potential difference) values are greater than 10 mV using the Eq. (2)^45^ and the results were incorporated in Table 1.

2 $$ \Delta {\text{Ep}} = 201.39\;\log \;\left( {\nu /k^{0} } \right) - 301.78 $$

**Table 1:** Variation of the voltammetric parameters for PA at different scan rates.

Scan rate (mVs^−1^)	ΔEp (mV)	k^0^ (s^−1^)
50	19	1.276
100	29	2.277
150	37	3.118
200	48	3.666
250	51	4.428
300	64	4.579
350	72	4.876
400	83	4.914
450	90	5.103
500	101	5.000

**Figure 10:**
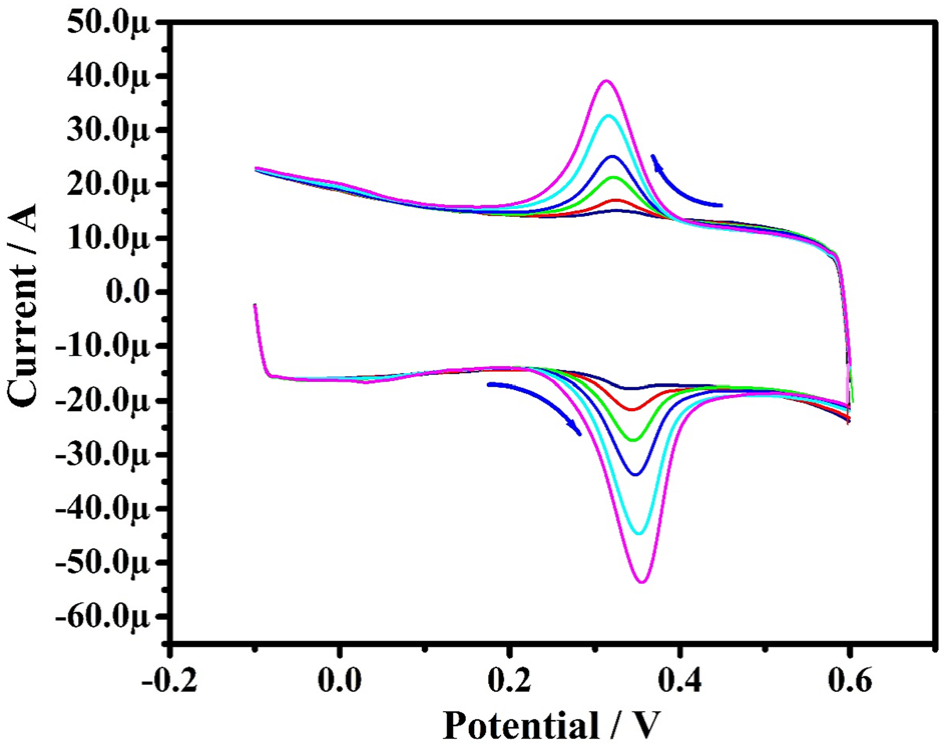
CVs for PA at different concentrations (10–60 µM) in PBS (0.2 M, pH 7.4) at PoOCD/MCPE.

**Figure 11:**
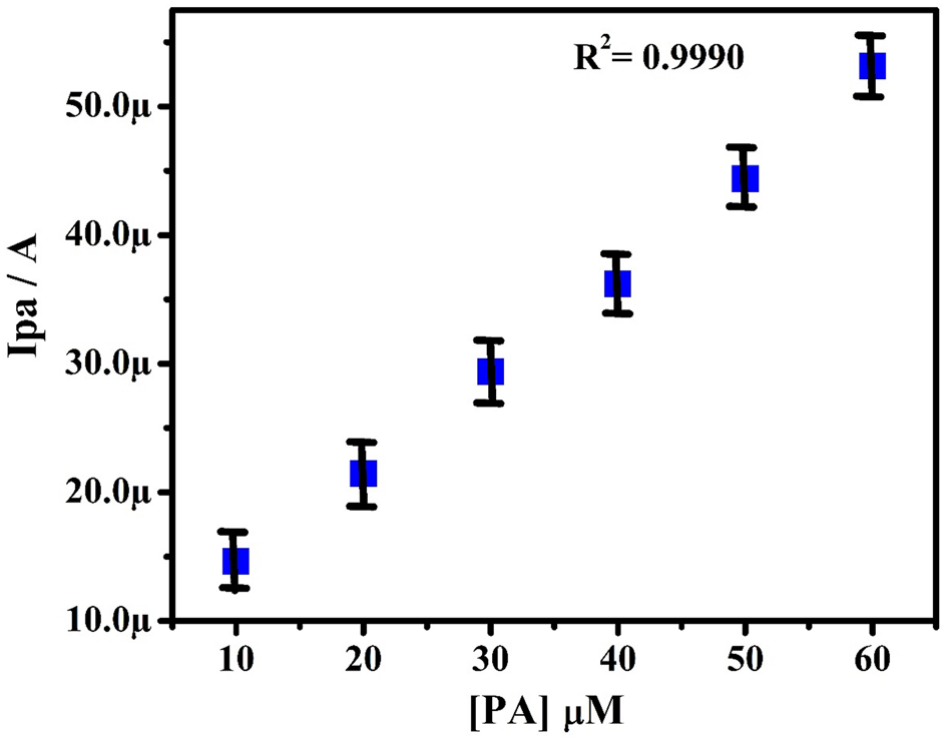
Graph of Ipa vs PA concentration.

The effect of PA concentration on redox behavior was studied at PoOCD/MCPE. Figure 10 depicts the CVs of 10–60 µM PA at PoOCD/MCPE in PBS (pH 7.4) at the SR of 50 mVs^−1^. By increasing PA concentration, the redox peak current gradually increased. Ipa vs PA concentration (Fig. 11) plot shows good linearity with regression equation Ipa (µA) = 0.7 (µM) + 6.38 (µA) (R^2^ = 0.9990). LOD and LOQ were calculated according to the Eqs. (3) and (4)^6,46^ for PA were found to be 2.64 µM and 8.81 µM respectively. The LOD of this modified electrode for the estimation of PA in comparison to other reported electrodes is given in Table 2

3 $$ {\text{LOD}} = 3{\text{S}}/{\text{M}} $$

4 $$ {\text{LOQ}} = 10{\text{S}}/{\text{M}} $$

where S is the standard deviation, M is the slope.

**Table 2:** Comparisons of the LOD of PoOCD/MCPE with other modified electrode reported.

Sl. no.	Electrode	Limit of detection (μM)	Method	References
1	Poly-NA-MCPE	7.2	CV	^2^
2	Diacerein/MCPE	3.8	DPV	^47^
3	N-DHPB-MWNT/CPE	10.0	DPV	^48^
4	Pd/Al	50.0	DPV	^49^
5	C_60_/GCE	50.0	DPV	^50^
6	Cu-poly-TTC	5.0	CV	^51^
7	PVA-Fe3O4/MGCE	8.0	DPV	^52^
8	GrRAC sensor	8.36	DPV	^53^
9	TiO_2_ nanoparticle MCPE	5.25	CV	^54^
10	PoOCD/MCPE	2.64	CV	This work

**Figure 12:**
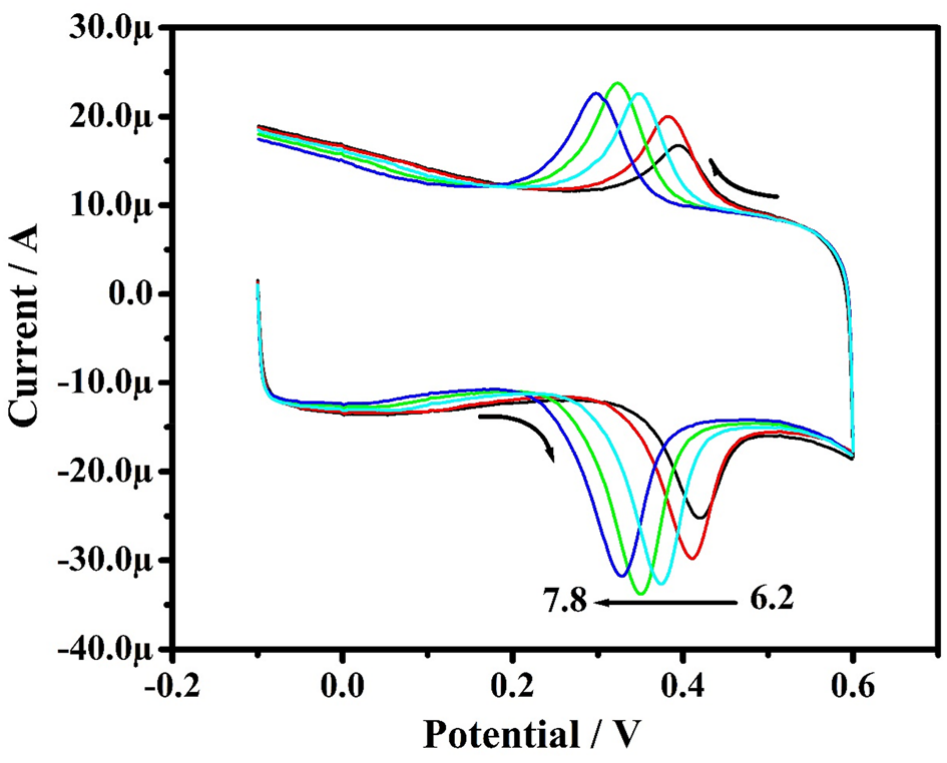
CVs for PA with varied pH at PoOCD/MCPE.

**Figure 13:**
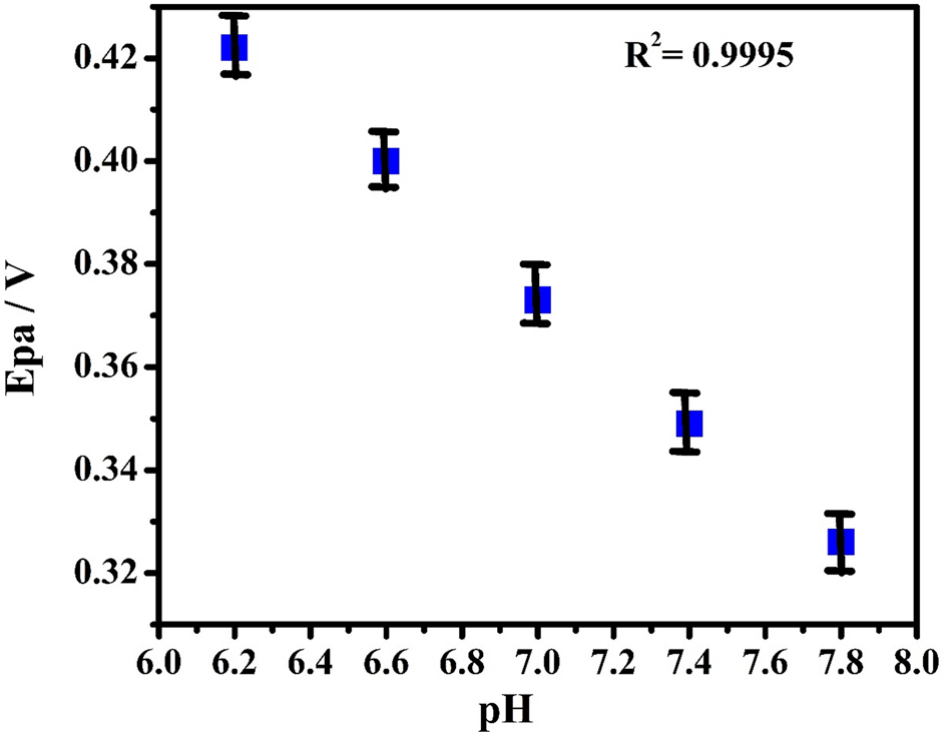
Graph of Epa vs varied pH for PA.

**Scheme 2.**
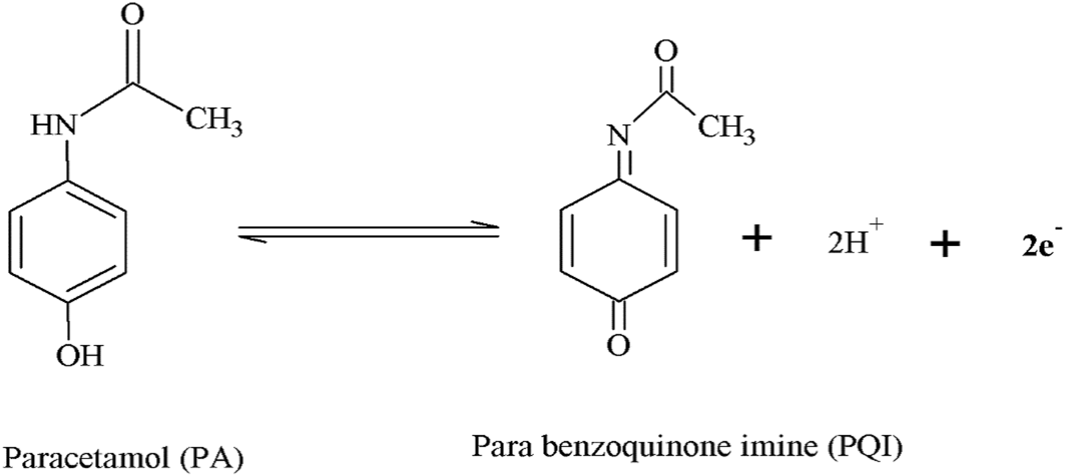
Oxidation mechanism of PA.

The pH plays a remarkable role in asses the number of participating electrons and protons in the oxidation mechanisms of the PA. The increase of pH (6.2–7.8) over PA (10 µM) oxidation at PoOCD/MCPE shifts Epa towards a more negative direction as analyzed by CV are shown in Fig. 12. Figure 13 illustrates the Epa vs pH values of PA graph that are linear with a slope of 0.0601 V/pH (R^2^ = 0.995). This suggests that during the oxidation of the PA, the same number of protons and electrons are participated^3,47^ and the possible electrooxidation was shown in Scheme 2.

### Simultaneous resolution of analytes PA, FA and DA

**Figure 14:**
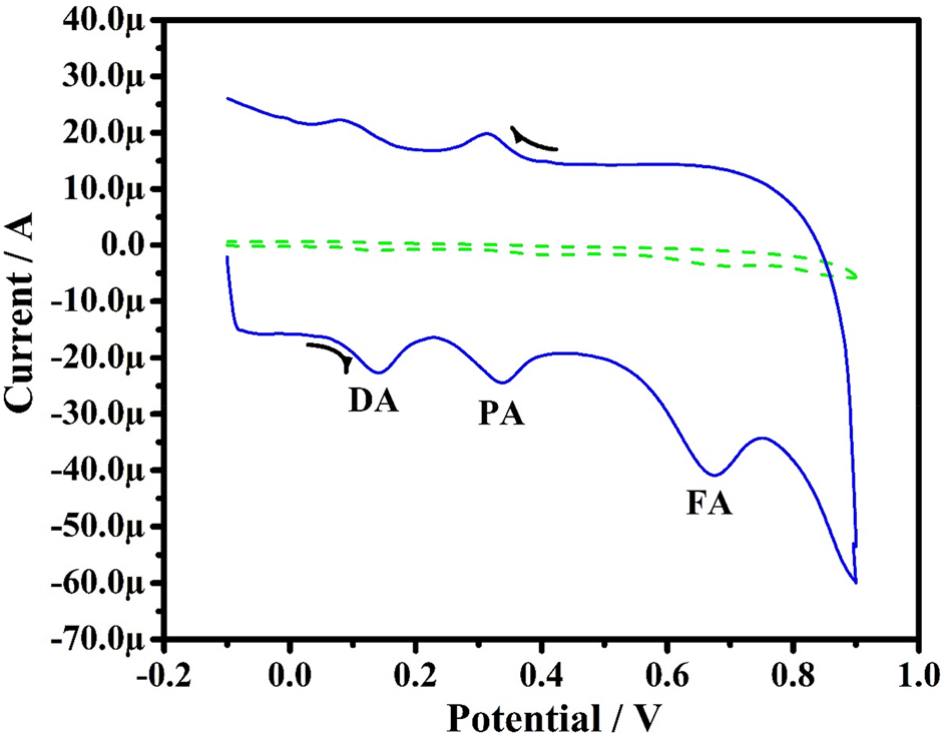
CV obtained for simultaneous studies of PA (0.1 M), FA (0.1 M) and DA (0.1 M) on bare CPE (**A**) and PoOCD/MCPE (**B**).

This study aimed to utilize the developed sensor for the selective and sensitive estimation of PA in the existence of FA and DA. Figure 14 illustrates the CVs recorded for the equimolar mixture (0.1 mM) of analytes PA, FA, and DA in 0.2 M PBS (pH 7.4) at SR 50 mVs^−1^ at bare CPE (A) and PoOCD/MCPE (B). At bare CPE, a low current signal with poor sensitivity was observed. However, in the same condition, the PoOCD/MCPE has shown a higher current signal with improved sensitivity for oxidation of DA, PA, and FA at 0.134 V, 0.408 V, and 0.695 V respectively. Hence, the developed PoOCD/MCPE serves as an excellent sensor for the PA.

### Interference studies

**Figure 15:**
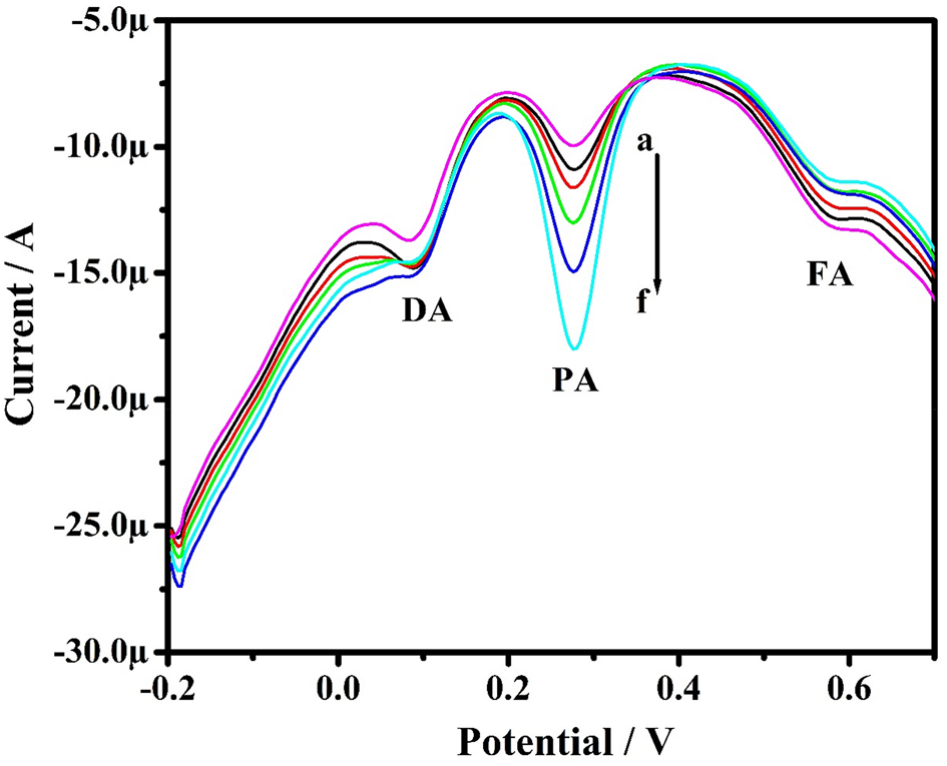
DPVs for PA at different concentrations (10–60 µM) in PBS (0.2 M, pH 7.4) at SR 50 mVs^−1^ at PoOCD/MCPE.

**Figure 16:**
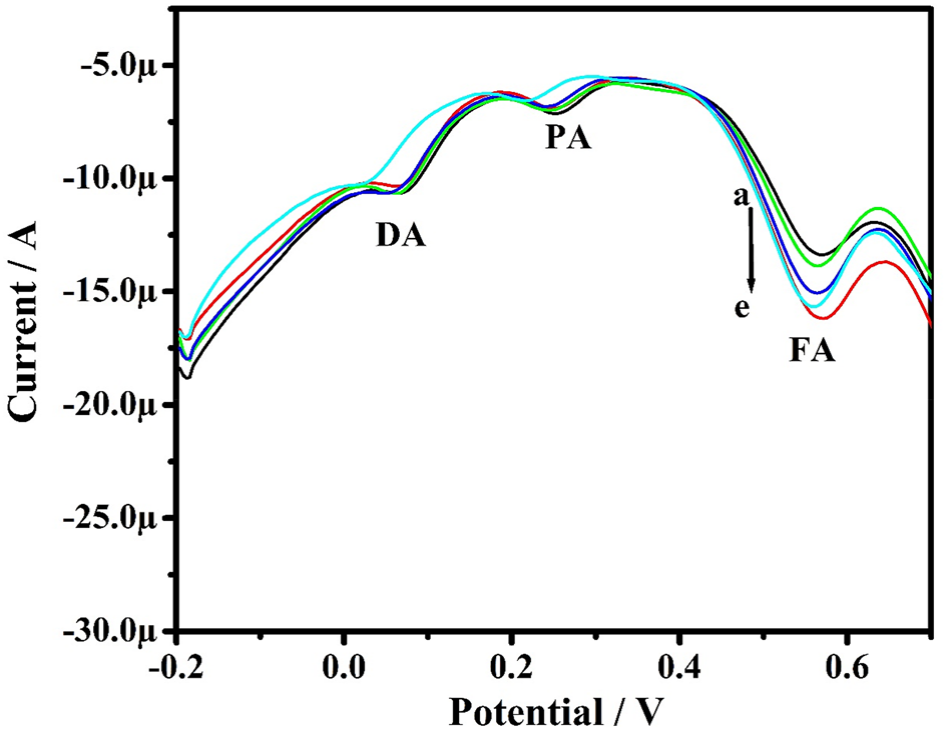
DPVs for FA at different concentrations (10–50 µM) in PBS (0.2 M, pH 7.4) at SR 50 mVs^−1^ at PoOCD/MCPE.

**Figure 17:**
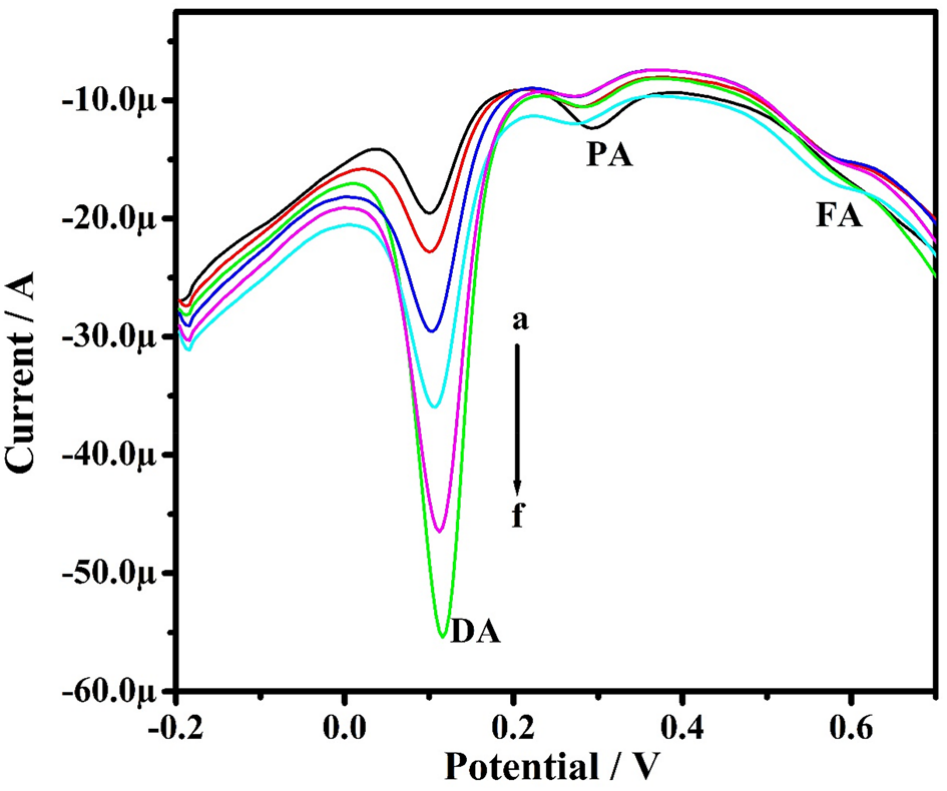
DPVs for DA at different concentrations (10–60 µM) in PBS (0.2 M, pH 7.4) at SR 50 mVs^−1^ at PoOCD/MCPE.

Studies were conducted by the DPV method in the solution mixture containing PA, FA, and DA at PoOCD/MCPE. The concentration of one analyte was varied, whereas the others were kept constant. Figure 15 illustrates the DPVs of PA by increasing the concentration of PA from 10 to 60 µM when holding the concentration of FA and DA constant. The oxidation peak current of PA increased linearly with increasing PA concentration from 10 to 60 µM and anodic peak current for FA and DA remaining constant. Similarly, it was also observed that the peak potentials remain unaltered with any enhancement in the peak current for the other two analytes. Figures 16 and 17 self illustrates the DVPs of FA (from 10 to 50 µM) and DA (from 10 to 60 µM) by keeping the other two analytes constant. These observations reveal that the oxidation of PA, FA, and DA has negligible influence on the variation of the other analytes. Therefore, PoOCD/MCPE showed good selectivity and sensitivity for the resolution of PA, FA, and DA.

### Repeatability, reproducibility and stability study

The repeatability of the PoOCD/MCPE for 0.1 mM PA in 0.2 M PBS (pH 7.4) was examined through five successive measurements and the RSD value of 2.3% demonstrates the superior repeatability of the MCPE. The reproducibility of the MCPE was investigated by fabricating five different MCPE under the same conditions. The RSD value obtained to be 4.8% confirms the good reproducibility. The stability of the MCPE was studied by 15 successive cycles (data not shown) remained 98% of its original current response for PA even after 15 cycles shows the good stability of the PoOCD/MCPE.

### Determination of PA in tablet sample

To evaluate the efficacy of PoOCD/MCPE in practical analysis, PA was successfully determined in tablet (Calpol 500 mg) by the CV method. The recovery test was done using the standard addition technique and the obtained results for four consecutive PA concentrations in the range from 10 to 40 μM were tabulated in Table 3. The acceptable percentage recoveries in the range of 98.28 ± 0.985 to 99.81 ± 0.545 obtained specify that the proposed sensor might be enough for practical application and can be employed for the determination of PA in pharmaceutical formulations.

**Table 3:** Evaluation of PA in tablet using PoOCD/MCPE.

Content	Added (µM)	Found (µM)	Recovery (%)
500 mg paracetamol tablet	10	9.9394	99.39 ± 0.125
	20	19.6563	98.28 ± 0.985
	30	29.8751	99.58 ± 0.315
	40	39.9242	99.81 ± 0.545

## Conclusion

This article reports the fabrication of novel, simple, sensitive and less cost sensor PoOCD/MCPE for voltammetric resolution of PA. The sensor shows high sensitivity, selectivity, and anti- interference capability for the electrochemical oxidation of PA. The developed PoOCD/MCPE displayed well separated and resolved peaks for the electro-oxidation of PA, FA, and DA. The sensor can be used for determining the PA individually and simultaneously in the existence of FA and DA. The capability of the sensor was studied by estimating PA in the tablet. The developed sensor can also be applied to estimate some other biomolecules in the pharmaceutical industry.
